# Using optogenetics to dissect rod inputs to OFF ganglion cells in the mouse retina

**DOI:** 10.3389/fopht.2023.1146785

**Published:** 2023-03-06

**Authors:** Asia L. Sladek, Wallace B. Thoreson

**Affiliations:** ^1^Truhlsen Eye Institute and Department of Ophthalmology and Visual Sciences, University of Nebraska Medical Center, Omaha, NE, United States; ^2^Pharmacology and Experimental Neuroscience, University of Nebraska Medical Center, Omaha, NE, United States

**Keywords:** rods, cones, optogenetics, retina, synaptotagmin, rod pathways

## Abstract

**Introduction:**

Light responses of rod photoreceptor cells traverse the retina through three pathways. The primary pathway involves synapses from rods to ON-type rod bipolar cells with OFF signals reaching retinal ganglion cells (RGCs) *via* sign-inverting glycinergic synapses. Secondly, rod signals can enter cones through gap junctions. Finally, rods can synapse directly onto cone OFF bipolar cells.

**Methods:**

To analyze these pathways, we obtained whole cell recordings from OFF-type α RGCs in mouse retinas while expressing channelrhodopsin-2 in rods and/or cones.

**Results:**

Optogenetic stimulation of rods or cones evoked large fast currents in OFF RGCs. Blocking the primary rod pathway with L-AP4 and/or strychnine reduced rod-driven optogenetic currents in OFF RGCs by ~1/3. Blocking kainate receptors of OFF cone bipolar cells suppressed both rod- and cone-driven optogenetic currents in OFF RGCs. Inhibiting gap junctions between rods and cones with mecloflenamic acid or quinpirole reduced rod-driven responses in OFF RGCs. Eliminating the exocytotic Ca^2+^ sensor, synaptotagmin 1 (Syt1), from cones abolished cone-driven optogenetic responses in RGCs. Rod-driven currents were not significantly reduced after isolating the secondary pathway by eliminating Syt1 and synaptotagmin 7 (Syt7) to block synaptic release from rods. Eliminating Syt1 from both rods and cones abolished responses to optogenetic stimulation. In Cx36 KO retinas lacking rod-cone gap junctions, optogenetic activation of rods evoked small and slow responses in most OFF RGCs suggesting rod signals reached them through an indirect pathway. Two OFF cells showed faster responses consistent with more direct input from cone OFF bipolar cells.

**Discussion:**

These data show that the secondary rod pathway supports robust inputs into OFF α RGCs and suggests the tertiary pathway recruits both direct and indirect inputs.

## Introduction

As light levels rise, the vertebrate retina transitions from relying on rod photoreceptor cells sensitive to dim lights to cone photoreceptor cells that respond to brighter lights. This transition involves a shift in the circuits used to transmit information from rods and cones to the output cells of the retina, retinal ganglion cells (RGCs). Signals from rod photoreceptor cells traverse the retina through at least three different pathways (1–3). The primary pathway operating at low light levels near scotopic threshold involves synapses from rods to ON-type rod bipolar cells (4, 5). Rod bipolar cells make glutamatergic synapses onto AII amacrine cells that in turn transmit signals to ON cone bipolar cells *via* gap junctions and to OFF cone bipolar cells *via* sign-inverting synaptic glycinergic synapses. The primary OFF pathway can therefore be blocked with a glycine receptor antagonist strychnine. A second pathway emerges at higher intensities whereby rod signals enter neighboring cones *via* transmission through gap junctions (6–11). There is evidence for this secondary rod pathway in human flicker ERG responses (12, 13). The secondary pathway can be inhibited by gap junction blockers like mecloflenamic acid (MFA). At still higher intensities, rods can use a third pathway involving direct synaptic contacts between rods and types 3 and 4 cone OFF bipolar cells (8, 11, 14–20). While the latter two pathways operate primarily at higher light levels, recent work suggests that all three pathways can help to shape rod responses even under dim light conditions (10). Other rod pathways that have been identified include glycinergic synapses from AII amacrine cells to specific ganglion cells (21) and synapses from rods to cone ON bipolar cells (22). However, this latter pathway is absent from rabbit retina (20).

In the present study, we combined optogenetic stimulation of rods and cones along with genetic elimination of exocytotic calcium sensors and gap junctions to distinguish the pathways carrying rod and cone signals to OFF α ganglion cells. We were interested in evaluating the strength of synaptic inputs entering α ganglion cells *via* the tertiary pathway involving direct contacts between rods and cone OFF bipolar cells. We expressed channelrhodopsin-2 (ChRh2) in rods and/or cones, allowing us to drive these two cell types independently with good temporal precision. Optogenetic activation of ChRh2 evokes depolarizing responses in rods or cones whereas activation of endogenous opsins evokes hyperpolarizing responses. Optogenetic activation therefore evokes inward currents in OFF cells at light onset rather than outward currents evoked by activation of endogenous opsins in rods and cones.

α RGCs are the most sensitive RGCs in mouse retina (23, 24). Both OFF transient and OFF sustained α ganglion cells receive glycinergic synaptic inputs from AII amacrine cells along with direct inputs from types 3 and 4 OFF cone bipolar cells. OFF sustained α RGCs receive stronger input from AII amacrine cells than OFF transient α RGCs (25, 26). Conversely, ultrastructural studies indicate that OFF transient α RGCs receive 40% of their input from Type 3A bipolar cells and 18% from Type 4 whereas OFF sustained α cells receive only 5% of their inputs from Type 3A and 4% from Type 4 (27, 28). We targeted cells by whole cell recording in flatmount retina and used optogenetics to analyze rod and cone inputs. Our results showed that with strong optogenetic stimulation, most OFF α RGCs receive strong input from primary and secondary pathways. Our results further suggest that signals entering the tertiary pathway reach most OFF α RGCs through a poly-synaptic pathway involving amacrine cells, but a subset of OFF cells receive fast rod input *via* direct synapses from cone OFF bipolar cells.

## Materials and methods

### Mice

Control and mutant mice were bred on C57/Bl6J backgrounds. Mice were kept on 12 hour dark-light cycles. Mice aged 6-12 weeks of both sexes were used for experiments. Rho-iCre, *HRGP-Cre*, *Syt1^flox^* (Syt1: MGI:99667), and Syt7^flox^ mice have been described previously (29–32). Ai32 mice that express channelrhodopsin2/EYFP fusion protein in the presence of cre-recombinase were obtained from Jackson Labs. *Rho-iCre* (RRID : IMSR_JAX:015850) mice were also obtained from Jackson Labs (30). Cx36 KO mice were generously provided by Eduardo Solessio (SUNY-Upstate) (33). To eliminate Syt1 and Syt7 from rods and cones, we crossed Rho-iCre and HRGP-Cre mice with Syt1^fl/fl^ and Syt7^fl/fl^ mice. To create mice that we could study optogenetically and lacked gap junctions between rods and cones, we crossed Rho-iCre mice with Ai32 and Cx36 KO mice (5). In our hands, homozygous Cx36 KO mice did not breed well making this a lengthy endeavor.

Animal care and handling protocols were approved by the University of Nebraska Medical Center Institutional Animal Care and Use Committee. Euthanasia was conducted in accordance with AVMA Guidelines for the Euthanasia of Animals by CO_2_ asphyxiation followed by cervical dislocation.

### Electrophysiology

Mice were dark-adapted overnight. After euthanasia, retinas were isolated under dim red light and then incubated in Ames’ medium supplemented with collagenase and hyaluronidase (Sigma-Aldrich) at room temperature for 15–30 min to aid in penetrating the inner limiting membrane (34). Retinas were placed with ganglion cells facing up in the recording chamber and held in place with a tissue slice anchor (Warner Instruments). Recordings were conducted under room light. The combination of room light and repeated LED stimulation placed the retina in a light-adapted condition.

Tissue was superfused at 3 ml/min. with Ames’ medium bubbled with 95% O_2_/5% CO_2_. Except where noted, we blocked glycine receptors with strychnine (1 μM). During ganglion cell recording, GABA receptors were inhibited by supplementing Ames medium with picrotoxin (100 μM) or gabazine (10 μM). Early experiments used gabazine but later studies–including pharmacological and gene knockout studies–used picrotoxin. While picrotoxin is more effective than gabazine in blocking GABA_c_ receptors (35), we saw no obvious differences in amplitude or kinetics of optogenetically-evoked currents of RGCs with these two compounds. Other pharmacological agents were also bath applied. Every experimental condition was repeated in RGCs from at least three different mice.

Whole cell recordings were obtained on an upright fixed-stage microscope (Olympus BX51) under a water-immersion objective (40x or 60x). Recording electrodes were fabricated from borosilicate glass pipettes (1.2 mm outer diameter, 0.9 mm inner diameter, World Precision Instruments) to yield a tip resistance of 5–7 MΩ. Pipettes were filled with solution containing (in mM): 110 Cs gluconate, 8 NaCl, 1 CaCl_2_, 20 BAPTA EGTA, 10 HEPES, 4 ATP, 0.01 Alexa 488, 5 QX314 (pH 7.4; 290 mOsm). The use of BAPTA eliminated retrograde signaling effects (36).

Recordings were performed in voltage clamp using an Axopatch 200B amplifier (Axon Instruments/Molecular Devices) and digitized with an ITC-18 interface (Heka Instruments). Data were acquired with AxoGraph X acquisition software and analyzed with Clampfit (Axon Instruments). Membrane currents were filtered at 5 kHz. RGCs were held at -60 mV. Voltages were not corrected for liquid junction potentials (Gluconate pipette solution: 12 mV).

ChR2 was activated by a 1 ms pulse of 490 nm light from an LED (Lambda TLED, Sutter Instruments). The voltage driving the LED was regulated by a computer-controlled analog input. For experiments reported here, we chose a voltage that consistently generated saturating responses (5 V).

Confocal images were obtained using Nikon Elements software and a laser confocal scanhead (Perkin Elmer Ultraview LCI) equipped with a cooled CCD camera (Hamamatsu Orca ER) mounted on a Nikon E600FN microscope. Fluorescent excitation was delivered from an argon/krypton laser at 488, 568, or 648 nm wavelengths and emission was collected at 525, 607, and 700 nm, respectively. Filters were controlled using a Sutter Lambda 10–2 filter wheel and controller. The objective (60X water immersion, 1.0 NA) was controlled using a E662 z-axis controller (Physik Instrumente). Image contrast and brightness was adjusted using Nikon Elements and Adobe Photoshop software.

### Statistical analysis

Statistical analysis and data visualization were done using GraphPad Prism 9. Data were analyzed with paired and unpaired t-tests, as well as ordinary one-way ANOVA. We adjusted for multiple comparisons using the Šídák method. The criterion for statistical significance was set at α = 0.05. Data in the text and figures are reported as mean ± SD.

## Results

**Figure 1:**
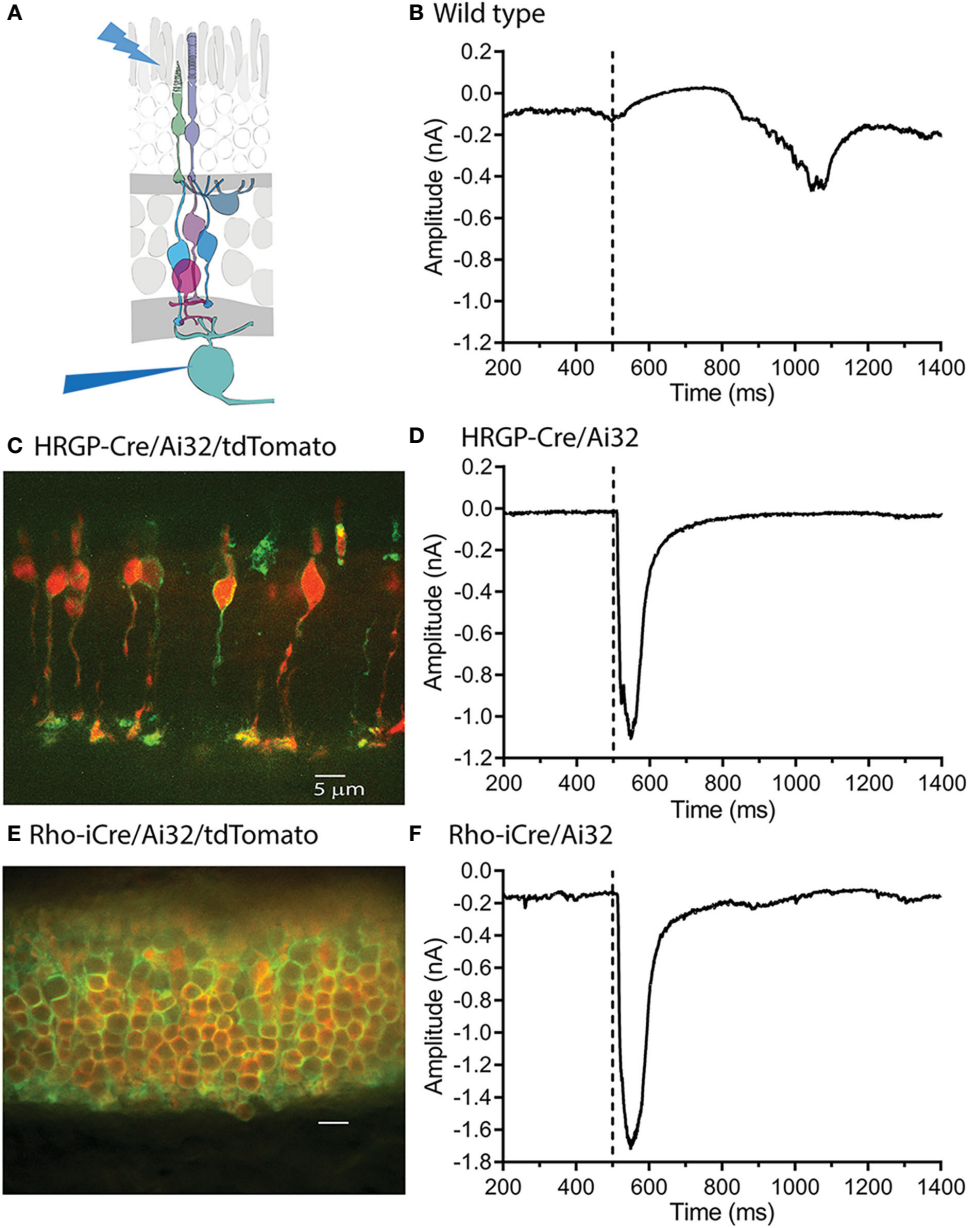
Optogenetic stimulation of ChRh2 expressed in rods or cones evoked large fast currents in OFF α ganglion cells. **(A)** Diagram illustrating the experimental protocol using blue light to stimulate rods and/or cones while recording from retinal ganglion cells. **(B)** Blue light stimuli evoke slow outward light responses in control C57 mice. These can be readily distinguished from fast inward currents evoked by optogenetic activation of ChRh2. **(C)** ChRh2 coupled to EYFP labeled cone membranes (green) in this retinal cross-section (maximum intensity projection) from an HRGP-Cre/Ai32 mouse. Cones in this retina were also labeled by Cre-dependent expression of td-Tomato (red). **(D)** An example of current evoked in an OFF RGC by optogenetic stimulation of cones. **(E)** EYFP labeled rod membranes (green) and td-Tomato labeled (red) rod cytoplasm in this retinal cross-section (maximum intensity projection) from a Rho-iCre/Ai32 mouse. **(F)** Current evoked in an OFF RGC by optogenetic stimulation of rods. Experiments in this figure were conducted in the presence of strychnine (1 μM) and gabazine (10 μM).

To compare rod and cone inputs into ganglion cells, we used Ai32 mice that express a ChRh2/EYFP fusion protein in the presence of Cre-recombinase. To express ChRh2 in rods or cones, we crossed these mice with Rho-iCre or HRGP-Cre mice, respectively. We used blue LED light to activate ChRh2 in photoreceptor cells and recorded synaptic currents in RGCs using a flatmount retinal preparation (**Figure 1A**). In the absence of Cre-recombinase, bright blue LED light typically evoked outward currents followed by slow inward currents in OFF-type ganglion cells (**Figure 1B**). These currents were evoked by hyperpolarizing cone responses that result from the activation of endogenous opsins by blue light in light-adapted retinas.

As illustrated in **Figure 1**, slow, endogenous currents could be readily distinguished from the rapid inward currents evoked in OFF RGCs by optogenetic activation of ChRh2. **Figure 1C** shows a retinal cross-section with cone membranes labeled by EYFP co-expressed with ChRh2 (green). Cone cytoplasm was labeled by the Cre-reporter, tdTomato. **Figure 1D** shows an example of a rapid inward current evoked in an OFF α RGC by optogenetic stimulation of cones in an HRGP-Cre/Ai32 mouse retina. **Figure 1E** shows a retinal cross section with expression of EYFP/ChRh2 in rod membranes and tdTomato in rod cytoplasm of Rho-iCre x Ai32 mice. **Figure 1F** shows an example of the rapid inward current evoked in an OFF α RGC by optogenetic activation of ChRh2 in rods.

Optogenetic activation of ChRh2 depolarizes cones whereas activation of endogenous cone opsin hyperpolarizes cones. Optogenetic activation therefore evokes inward currents in OFF RGCs at light onset whereas activation of endogenous opsins evokes outward currents at light onset and inward currents at light offset (**Figure 1**) Endogenous responses were much slower than optogenetic responses but by countering later portions of inward optogenetic current, may have slightly reduced total charge transfer (compare the endogenous light response in **Figure 1B** with optogenetic responses in the lower panels).

**Figure 2:**
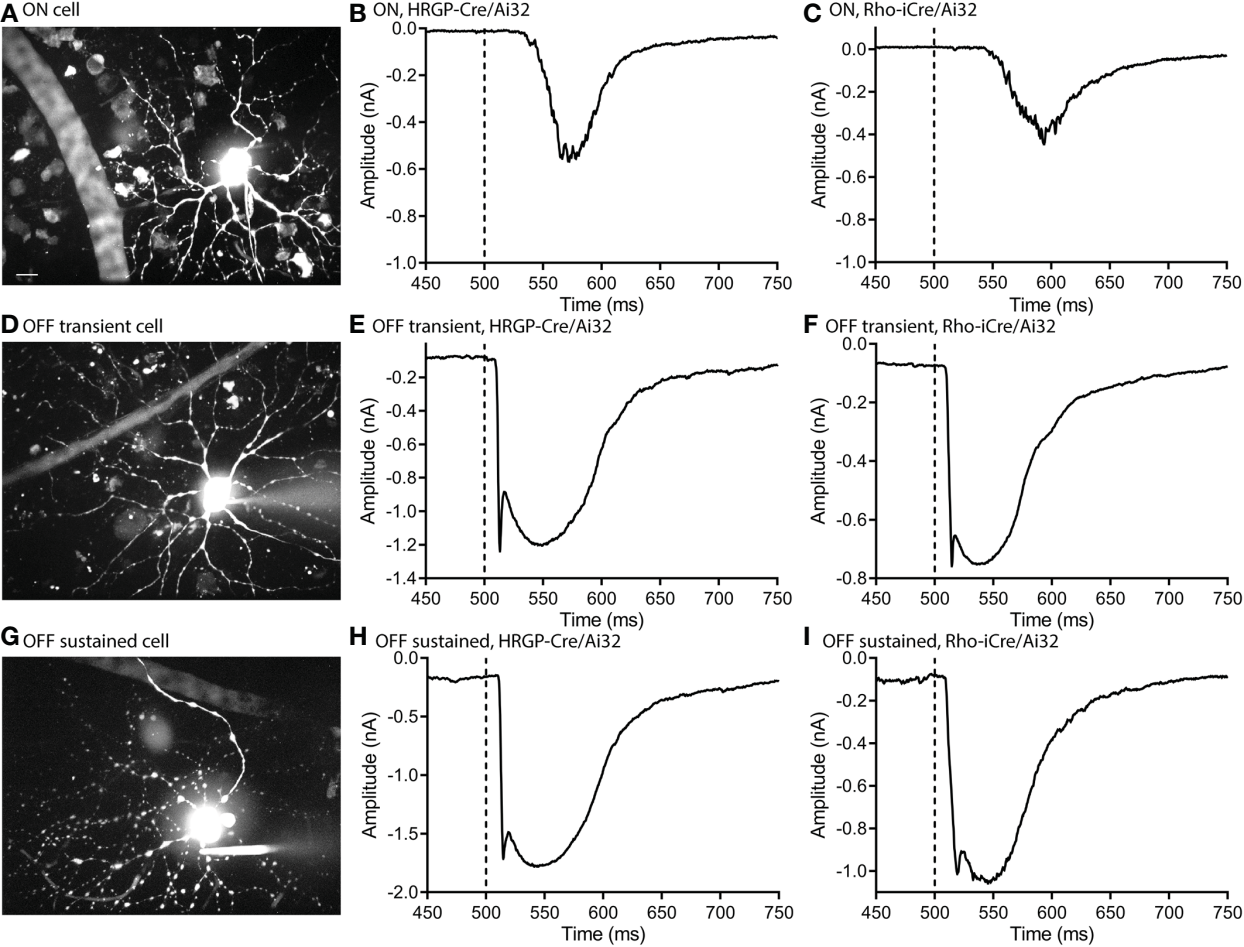
Optogenetic stimulation evoked similar fast inward currents in OFF transient and OFF sustained α retinal ganglion cells. ON cells showed slower inward currents. **(A)** Maximum intensity image of an ON α cell filled with Alexa 488. **(B)** Response of an ON α cell to optogenetic stimulation of cones showing a delayed inward current. **(C)** Response of an ON α cell to optogenetic stimulation of rods. **(D)** Maximum intensity image of an OFF transient α cell filled with Alexa 488. **(E)** Response of an OFF transient α cell to optogenetic stimulation of cones. **(F)** Response of an OFF transient α cell to optogenetic stimulation of rods. **(G)** Maximum intensity image of an OFF sustained α cell filled with Alexa 488. **(H)** Response of an OFF sustained α cell to optogenetic stimulation of cones. **(I)** Response of an OFF sustained α cell to optogenetic stimulation of rods. Experiments in this figure were conducted in the presence of strychnine (1 μM) and gabazine (10 μM).

Optogenetic stimulation of ON RGCs (**Figures 2A–C**) evoked smaller and slower inward currents than stimulation of OFF cells (**Figures 2D–I**). Glutamate release from rods in darkness keeps the signaling cascade activated by mGluR6 receptors in rod bipolar cells near saturation (37). This limits the impact of further depolarization in rods and thereby limits the outward currents that can be evoked by optogenetic stimulation of rods in ON RGCs. The only optogenetic responses visible in these ON cells were therefore OFF responses (**Figures 2B, C**).

**Figure 3:**
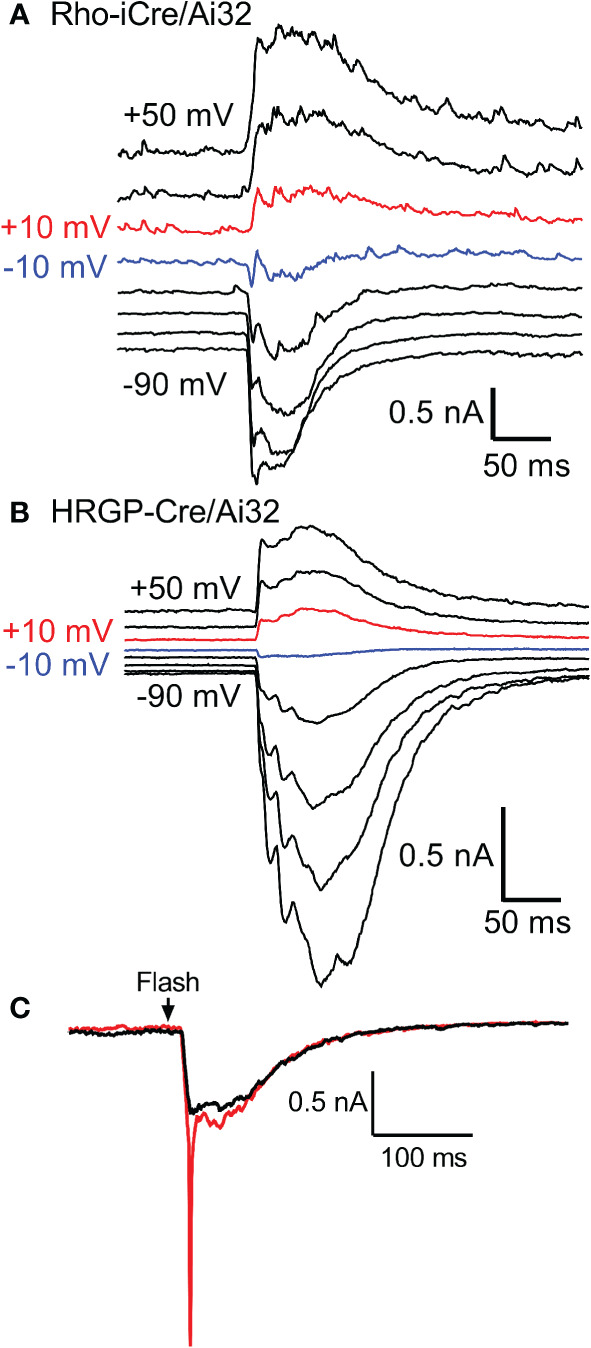
Responses of OFF α RGCs at different membrane potentials (20 mV steps from -90 to +50 mV) evoked by optogenetic stimulation of rods **(A)** and cones **(B)**. The recording in A was from an OFF transient cell in a Rho-iCre/Ai32 retina and the recording in B was from an OFF sustained cell in HRGP-Cre/Ai32 retina. In both, responses reversed between -10 mV (blue trace) and +10 mV (red trace). **(C)** At the beginning of a recording, optogenetic stimulation often evoked a rapid inward current arising from voltage-dependent Na^+^ currents (red trace). Na^+^ currents disappeared during the first few minutes as the Na^+^ channel blocker QX314 (5 μM) diffused into the cell through the patch pipette (black trace). This example was from an OFF transient cell in an HRGP-Cre/Ai32 retina. Experiments in this figure were conducted in the presence of strychnine (1 μM) and gabazine (10 μM).

We targeted OFF α RGCs that receive rod and cone inputs for these experiments. In the presence of GABA antagonists (gabazine or picrotoxin) and the glycine receptor antagonist strychnine, responses of OFF cells to optogenetic stimulation of rods (n=8) and cones (n=12) reversed at positive potentials consistent with excitatory inputs. This is illustrated in **Figures 3A, B** with examples from rod- and cone-driven cells in retinas of Rho-iCre/Ai32 and HRGP-Cre/Ai32 mice. We could not use conventional light response criteria to classify sustained *vs*. transient cells. Instead, OFF transient α RGCs were distinguished from OFF sustained α RGCs by the presence of prominent low-voltage-activated T-type Ca^2+^ currents in the former (26, 36, 38, 39). We tested for T-type currents using a voltage step from -90 to -50 mV. We included the dye Alexa488 in the patch pipette to label cells for anatomical confirmation whenever possible. OFF transient α RGCs terminate in the proximal half of the inner plexiform layer whereas OFF sustained α RGCs terminate more deeply in the distal half (26, 36, 40, 41).

Excitatory synaptic currents evoked in OFF α RGCs by optogenetic stimulation of cones or rods typically consisted of both fast and slow components. In some cells, the initial fast component was only an inflection during the rising phase of the inward current (e.g., **Figure 1F**). We included a Na^+^ channel blocker QX314 in the recording pipette. Contributions of Na^+^ currents produced an abrupt acceleration of the initial inward optogenetic current. As illustrated by example responses in **Figure 3C**, Na^+^ currents declined during the first few minutes of recording as QX314 entered the cell through the patch pipette. When reporting amplitude or latency of peak inward currents we did not include current components that showed evidence of sodium channel contributions.

### Pharmacology of ganglion cell currents evoked by optogenetic stimulation of rods or cones

**Figure 4:**
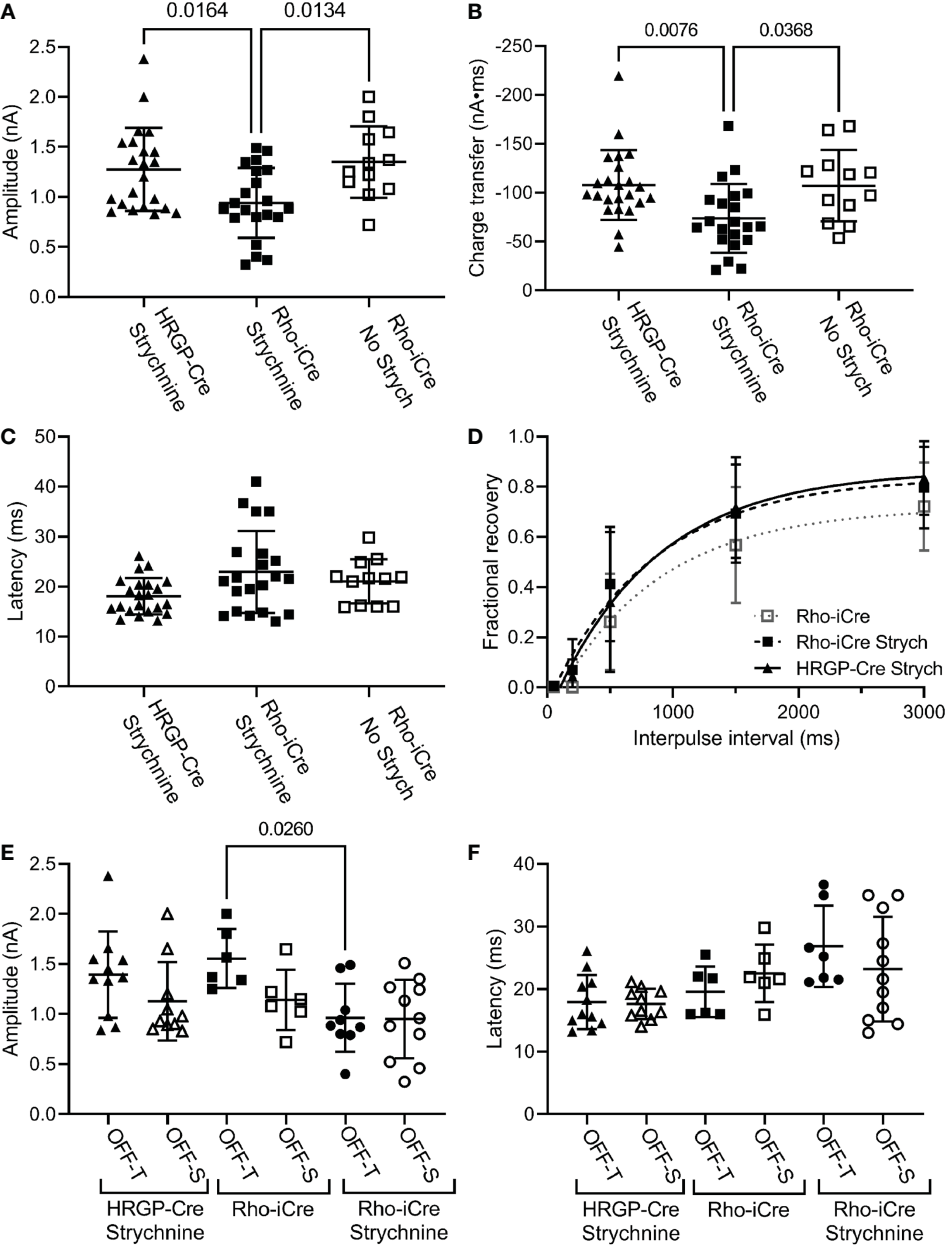
Comparison of rod and cone-driven optogenetic responses of OFF α RGCs. **(A)** Amplitude of currents evoked by optogenetic stimulation of rods was reduced significantly by the presence of strychnine (1 μM). (Rho-iCre with strychnine, n=21 cells, 9 mice; Rho-iCre without strychnine, n=12 cells, 5 mice; HRGP-Cre with strychnine, n=22 cells, 8 mice). **(B)** Charge transfer of c evoked by optogenetic stimulation of rods was also reduced significantly by the presence of strychnine. **(C)** Latency of rod-driven optogenetic currents was increased by an average of 4.8 ms compared to cone-driven optogenetic currents. **(D)** Recovery from paired pulse depression was similar for rod-driven optogenetic currents with (τ = 685 ms; df=94) and without strychnine (τ = 781 ms; df=57) and for cone-driven currents with strychnine (τ=852 ms, df=124). **(E)** Optogenetically-evoked current amplitude did not differ significantly between OFF-transient and OFF-sustained cells in both rod- and cone-driven cells. Strychnine reduced the amplitude of rod-driven currents in OFF transient cells significantly (Rho-iCre transient and sustained RGCs without strychnine, n=6 cells each,; Rho-iCre transient and sustained RGCs with strychnine, n=9 and n=11 cells, respectively; HRGP-Cre transient and sustained RGCs, n=11 and n=10 cells, respectively). OFF transient and sustained cells were distinguished by the presence or absence of prominent T-type currents and confirmed anatomically whenever possible. **(F)** Latency to the fast component of the optogenetic response did not differ significantly among rod- and cone-driven sustained and transient OFF α RGCs. Most experiments in this figure were conducted in the presence of strychnine (1 μM) and picrotoxin (100 μM). In early experiments, we used gabazine (10 μM) rather than picrotoxin.

We compared currents in OFF α RGCs driven by optogenetic stimulation of rods or cones. Optogenetic stimulation of rods in Rho-iCre/Ai32 mice evoked responses in ganglion cells with similar waveforms as cone-driven currents in HRGP-Cre/Ai32 mice (**Figure 2**). Early experiments used gabazine (10 μM) but most used picrotoxin (100 μM) to inhibit GABA receptors. We saw no differences in amplitude or kinetics and so combined data from these two compounds in our control sample. In the presence of strychnine and GABA antagonists, the peak amplitude of cone-driven currents averaged 1.23 ± 0.41 nA (SD, n=22 cells) whereas rod-driven currents were significantly smaller, averaging 0.91 ± 0.38 nA (n=21; p = 0.0272; **Figure 4A**). Charge transfer was also significantly greater for cone-driven (108.4 ± 37.3 nA*ms) than rod-driven currents (75.6 ± 33.6 nA*ms; p = 0.003; **Figure 4B**). Under these same conditions, the latency to the initial fast component for cone-driven currents averaged 18.1 ± 3.55 ms (n=22) whereas rod-driven currents showed a significantly longer latency of 22.9 ± 8.2 ms (n=21; p=0.04) (**Figure 4B**). Latency for rod-driven currents shortened from 18.1 ms at room temperature to 9.0 ± 2.95 ms (n= 4) at 35 deg C (paired t-test, t=3.315, df = 23; p=0.003).

In most of our experiments, the primary pathway from rod bipolar cells to OFF ganglion cells was blocked by using strychnine to inhibit glycinergic synapses between AII amacrine cells and OFF cone bipolar cells. To evaluate contributions of the primary pathway, we compared optogenetic stimulation of rods in Rho-iCre/Ai32 mice with and without strychnine. Strychnine (1 μM) reduced both amplitude (**Figure 4A**; no strychnine: 1.35 ± 0.36 nA, n=12; strychnine: 0.90 ± 0.38 nA, n=21; p=0.0112, one-way ANOVA with Sidak’s multiple comparisons test) and charge transfer (**Figure 4B**, 107.2 ± 36.6 nA*ms; strychnine: 75.6 ± 33.6 nA*ms; p=0.023) of rod-driven currents in OFF RGCs (**Figure 4B**). Latency was unchanged (**Figure 3C**). Blocking glycinergic inhibition could allow more sustained release of glutamate, but amplitude and charge transfer were both reduced by a similar fraction (0.33 and 0.30, respectively).

We found no differences in paired pulse depression between rod and cone-driven pathways. In the presence of strychnine, the recovery from paired pulse depression was similar whether release was driven by rods in Rho-iCre/Ai32 mice or cones in HRGP-Cre/Ai32 mice (**Figure 4D**). This is consistent with shared retinal circuits. Recovery was a bit slower when strychnine was omitted from Rho-iCre/Ai32 experiments, but the difference was not significant (**Figure 4D**).

The amplitude and latency to the initial peak current did not differ significantly between OFF-transient and OFF-sustained RGCs for either rod- or cone-driven cells (**Figures 4E, F**). The addition of strychnine had a greater impact in reducing the amplitude of rod-driven currents in OFF transient cells than OFF sustained cells (**Figure 4**). However, in the presence of strychnine, we saw no significant differences between OFF sustained and OFF transient cells in terms of amplitude or latency and so we combined data from these two cell types for most subsequent analyses.

**Figure 5:**
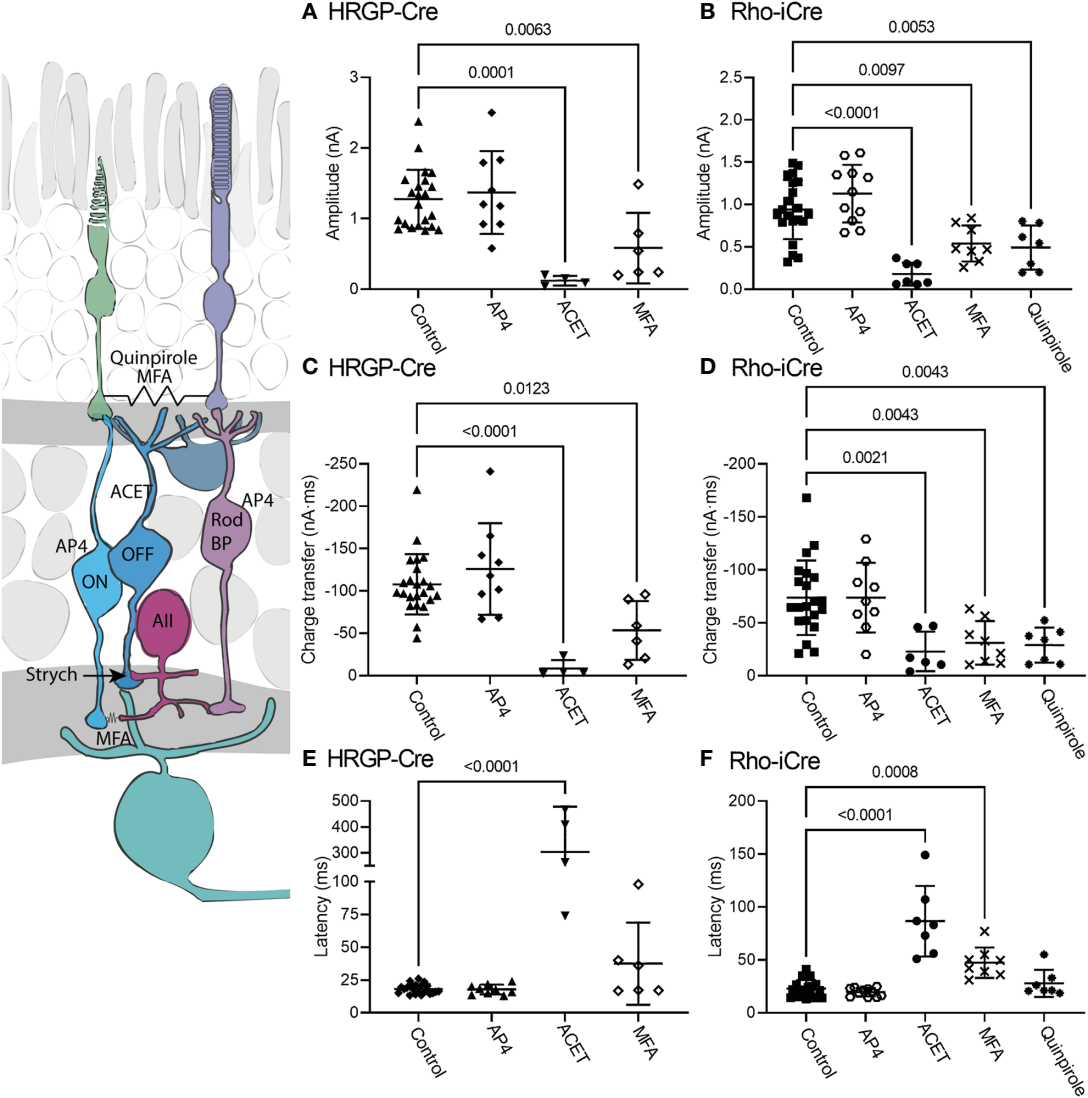
Pharmacological analysis of rod inputs to OFF α RGCs. At the left is a diagram illustrating the sites targeted pharmacologically. L-AP4 saturates mGluR6 glutamate receptors in rod and ON cone bipolar cells. ACET inhibits KA receptors in cone OFF bipolar cells. MFA targets rod-cone gap junctions and gap junctions between AII amacrine cells and ON cone bipolar cells. The D2/D4 dopamine receptor agonist quinpirole also reduces gap junctional coupling between rods and cones. Strychnine was present throughout these experiments to block the glycinergic synapse from AII amacrine cells to OFF cone bipolar cells that convey signals from rod bipolar cells into the OFF pathway. **(A)** Responses evoked by optogenetic stimulation of cones were reduced significantly by blocking KA receptors with ACET (10 μM, n=4 cells, 3 mice) or by using the gap junction blocker, MFA (100 μM, n=6 cells, 6 mice). (HRGP-Cre control, n=22 cells, 8 mice; HRGP-Cre AP4, n=9 cells, 3 mice) **(B)** Responses evoked by optogenetic stimulation of rods were reduced significantly by blocking KA receptors with ACET (10 μM, n=8 cells, 3 mice), the gap junction blocker, MFA (100 μM, n=8 cells, 8 mice), and the D2/D4 dopamine receptor agonist quinpirole (1-3 μM, n=7 cells, 7 mice). (Rho-iCre control, n=21; Rho-iCre AP4, n=9 cells, 6 mice). **(C)** Cone-driven response charge transfer was also significantly reduced by ACET and MFA. **(D)** Rod-driven response charge transfer was significantly reduced by ACET, MFA and quinpirole. **(E)** Latencies of responses evoked by optogenetic stimulation of cones were lengthened significantly by ACET. **(F)** Latencies of responses evoked by optogenetic stimulation of rods were lengthened significantly by ACET and MFA. Most experiments in this figure were conducted in the presence of strychnine (1 μM) and picrotoxin (100 μM). In some early experiments on Rho-iCre or HRGP-Cre mice, we used gabazine (10 μM) rather than picrotoxin.

The diagram at the left of **Figure 5** illustrates the different sites targeted pharmacologically. L-AP4 was used to saturate mGluR6 glutamate receptors in rod and ON cone bipolar cells, thereby blocking the primary rod pathway. Strychnine also inhibits the primary rod OFF pathway by targeting glycinergic synapses from AII amacrine cells to OFF cone bipolar cells. Inhibiting KA receptors in cone OFF bipolar cells with ACET should block both secondary and tertiary rod pathways. MFA targets rod-cone gap junctions as well as gap junctions between AII amacrine cells and ON cone bipolar cells, thereby blocking the secondary rod pathway. The D2/D4 dopamine receptor agonist quinpirole also reduces gap junctional coupling between rods and cones, thereby inhibiting the secondary pathway.

When added in the presence of strychnine, L-AP4 (20 μM) caused no further decrease in current amplitude or charge transfer (**Figure 5**). Strychnine was present for all experiments plotted in **Figure 4** and the negligible effects of adding L-AP4 supports the conclusion that strychnine alone successfully blocked rod bipolar cell inputs into OFF α RGCs conveyed by glycinergic synapses from AII amacrine cells to OFF cone bipolar cells. Additionally, the reduction in amplitude and charge transfer produced by strychnine alone or strychnine plus AP4 suggests that a third of the total OFF input when driven by optogenetic stimulation of rods involves glycinergic synapses.

**Figure 6:**
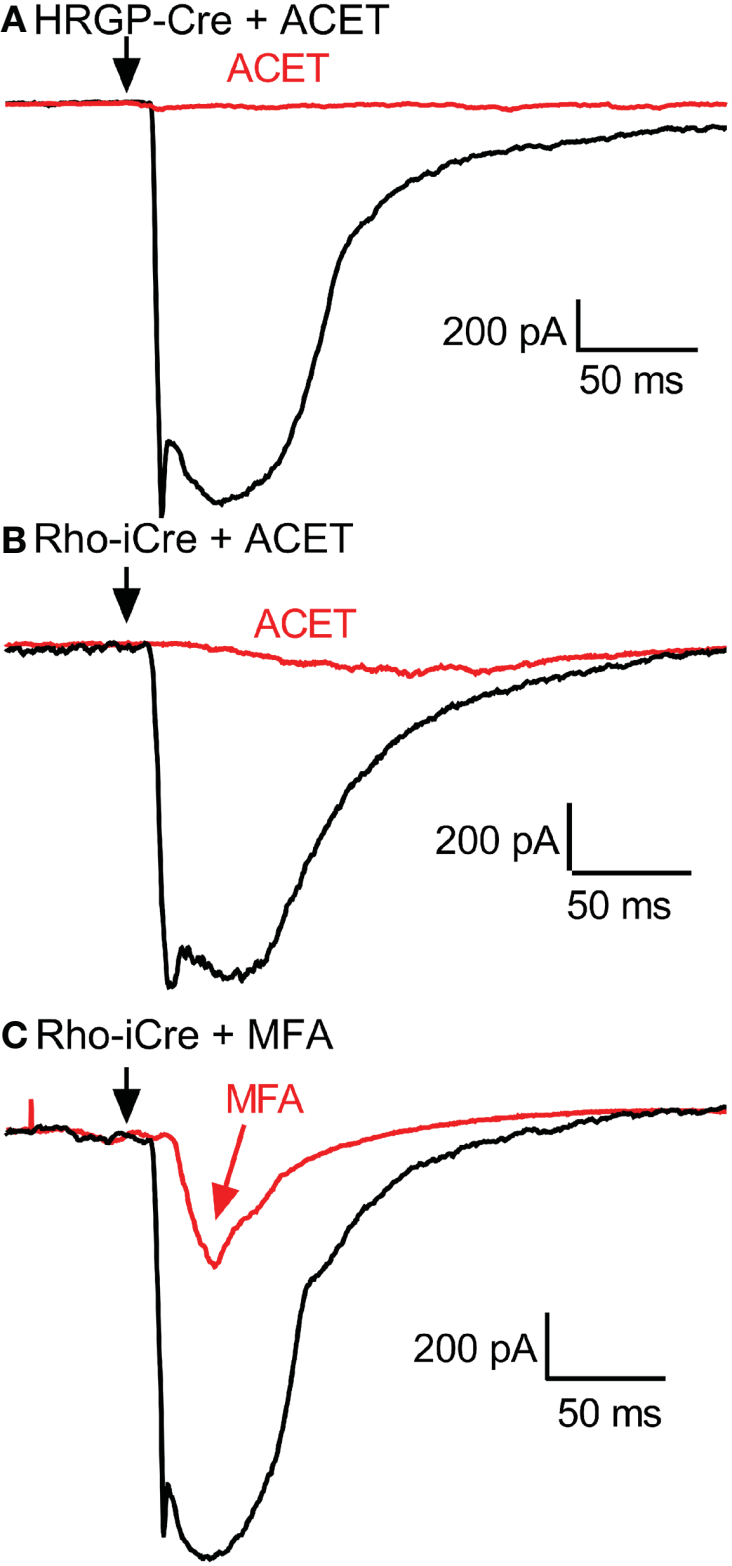
Example RGC response waveforms showing responses evoked by optogenetic stimulation of cones with and without ACET **(A)**, stimulation of rods with and without ACET **(B)**, and stimulation of rods with and without MFA **(C)**. Experiments in this figure were conducted in the presence of strychnine (1 μM) and picrotoxin (100 μM).

Consistent with other studies (42–47), we found that blocking KA receptors in OFF bipolar cells with ACET (10 μM) almost completely abolished optogenetically-evoked currents in OFF α RGCs, whether driven by rods or cones. The bar graphs in **Figure 5** summarize the changes in current amplitude (A, B), charge transfer (C, D), and latency to the initial peak (E, F). Representative waveforms are shown in **Figure 6**. Residual currents observed in the presence of ACET were considerably slower than those in control conditions. OFF α RGCs possess AMPA receptors and so they should be relatively immune to direct effects of ACET (48–51). The modest effects of strychnine together with the potent inhibitory effects of ACET on rod-driven currents in OFF RGCs suggests that most of the rod-driven currents observed in the presence of strychnine involve transmission to KA receptors at cone OFF bipolar cell dendrites. This could arise from secondary (rod to cone transmission *via* gap junctions) or tertiary rod pathways (direct rod inputs to cone OFF bipolar cells).

To examine contributions of rod-cone gap junctions to these currents, we tested effects of the gap junction blocker MFA (100 μM). Summary data are shown in **Figure 5** and waveforms obtained before and after application of MFA are illustrated in **Figure 6C**. After applying MFA, rod-driven currents rapidly lost an initial fast component and showed a significantly longer latency (P=0.0008, Sidak’s multiple comparisons test). The amplitude and charge transfer continued to diminish in the presence of MFA but currents were not fully abolished even after 10-20 min. Currents evoked in OFF RGCs by optogenetic stimulation of cones were also reduced in amplitude and charge transfer by MFA. This could potentially involve inhibition of cone signals traveling into rods but might also be due to non-specific effects of MFA. The latency for cone-driven currents was slowed by MFA but this effect was not statistically significant (**Figure 5**).

In a second approach to testing gap junction contributions to rod-driven currents, we applied the D2/D4 dopamine receptor agonist quinpirole (1-3 μM). Quinpirole has been shown to uncouple gap junctions between rods and cones (5). Like MFA, this drug produced a significant reduction in peak current amplitude (p=0.005, n=7) and charge transfer (p=0.0018) of rod-driven currents compared to control (strychnine + picrotoxin; **Figure 5**). Response latency was not significantly different from control. MFA and quinpirole have quite different pharmacological profiles and off-target effects. The reduction in optogenetic currents produced by both compounds supports the idea that the principal effects in both experiments involve inhibition of rod-cone gap junctions, suggesting a major role for rod-cone coupling in transmission of OFF responses to α RGCs. Are the residual currents in rod-driven cells due to incomplete block of gap junctions or the result of direct contacts between rods and cone OFF bipolar cells? To answer this question, we turned to studies using genetically modified mice.

### Genetic elimination of Syt1, Syt7, and Cx36

Syt1 appears to be the sole exocytotic Ca^2+^ sensor used by cones to mediate synaptic release of glutamate-filled vesicles. Syt1 also controls a fast form of release from rods and eliminating Syt1 from rods and cones completely abolishes ERG b-waves (31, 32). To eliminate Syt1 from cones or rods selectively, we crossed floxed Syt1 mice with mice expressing Cre-recombinase in cones (HRGP-Cre) or rods (Rho-iCre) (31).

**Figure 7:**
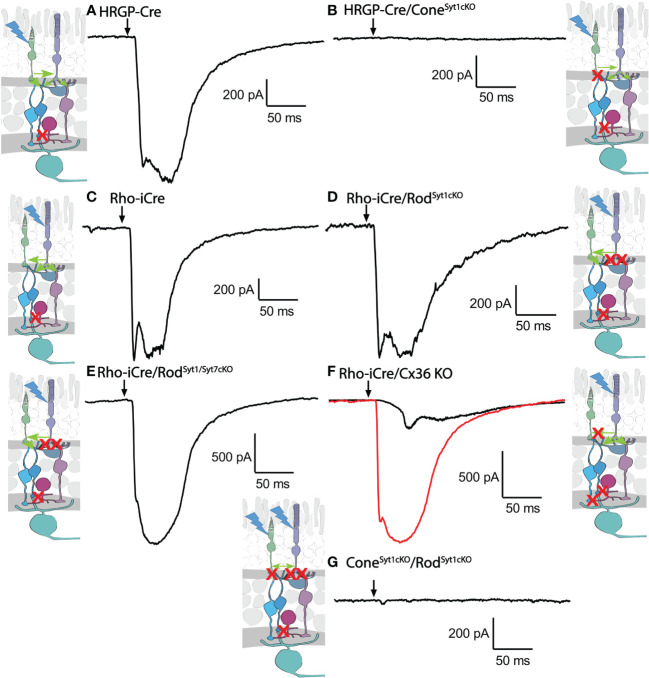
Example waveforms of responses evoked by optogenetic stimulation of rods and/or cones lacking Syt1, Syt7, or Cx36. **(A)** Current evoked by optogenetic stimulation of cones in an α RGC. Red X in the diagram represents blockade of glycinergic synapses from AII amacrine cells to cone OFF bipolar cells by strychnine. **(B)** Response to optogenetic stimulation of cones was abolished by the absence of Syt1 from cones. **(C)** Example current evoked by optogenetic stimulation of rods. **(D)** Response to optogenetic stimulation of rods was remained in the absence of Syt1 from rods. **(E)** Response to optogenetic stimulation of rods also remained in the absence of both Syt1 and Syt7 from rods. **(F)** Two example responses to optogenetic stimulation of rods in Cx36KO mice. Black trace shows the typical slow response that was observed in most cells. The red trace shows the large fast response observed in one OFF cell. **(G)** Response to optogenetic stimulation of rods and cones was abolished by the absence of Syt1 from both rods and cones. Experiments in this figure were conducted in the presence of strychnine (1 μM) and picrotoxin (100 μM).

**Figure 8:**
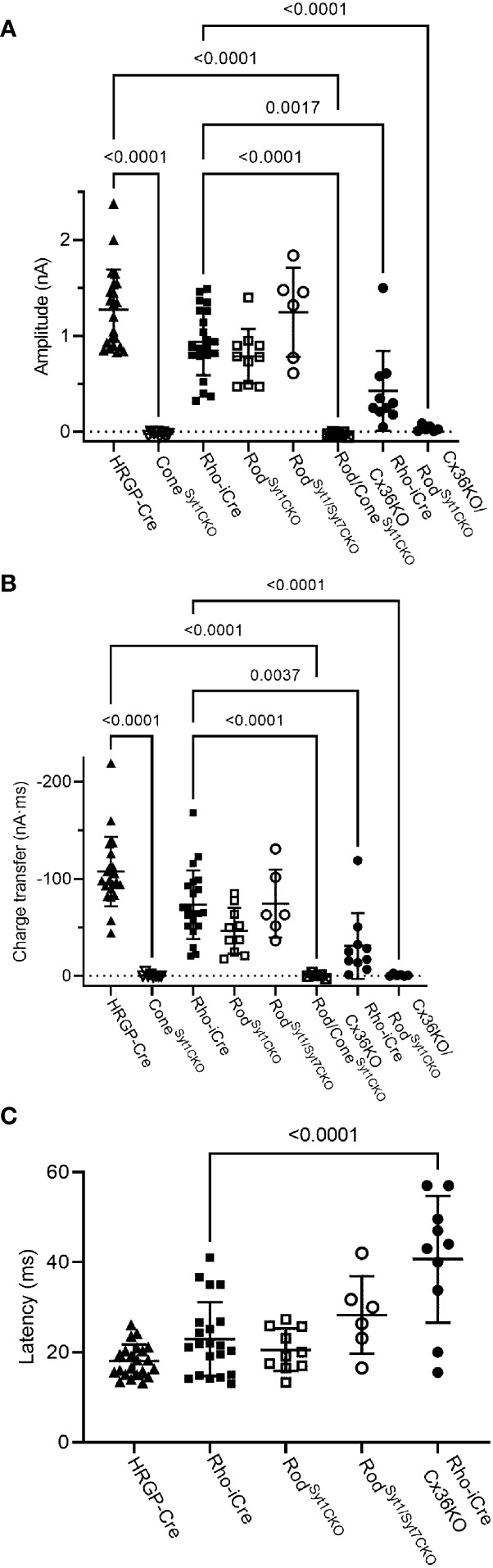
Summary of optogenetically-evoked responses in RGCs from different genotypes. **(A)** Response amplitude evoked by stimulation of rods or cones were both reduced significantly by elimination of Syt1 from cones. Eliminating Syt1 from rods did not significantly reduce responses evoked by optogenetic stimulation of rods. Eliminating Cx36 also significantly reduced responses evoked by optogenetic stimulation of rods. Residual responses in Cx36KO mice were abolished by eliminating Syt1 from rods. **(B)** Charge transfer measurements in the same cells showed similar effects. **(C)** Latency measurements showed that eliminating Cx36 lengthened the average latency of responses evoked by optogenetic stimulation of rods. Latencies were not measured in the three experimental conditions where responses were abolished. This included both genotypes lacking Syt1 in cones as well as Cx36KO retinas lacking Syt1 in rods. Cone^Syt1CKO^, n = 8 cells (6 mice); Rod^Syt1CKO^, n = 10 cells (7 mice); Rod^Syt1Syt7CKO^, n = 6 cells (2 mice); Rod/Cone^Syt1CKO^, n = 5 cells (5 mice); Rho-iCre/Cx36KO, n = 10 cells (3 mice). Experiments in this figure were conducted in the presence of strychnine (1 μM) and picrotoxin (100 μM).

Consistent with evidence that Syt1 is the sole sensor used by cones, eliminating Syt1 from cones in HRGP-Cre/Syt1^flfl^/Ai32- mice abolished responses in RGCs driven by optogenetic stimulation of cones. **Figure 7A** shows a control recording with Syt1 intact while **Figure 7B** shows a recording from an OFF α RGC after Syt1 was eliminated from cones. As summarized in **Figure 8**, eliminating Syt1 from cones consistently abolished cone-driven optogenetic responses in RGCs, assessed by both amplitude and total charge transfer.

Rods use Syt1 for fast, synchronous release of glutamate and eliminating Syt1 suppresses scotopic ERG b-waves (31, 32). However, genetically eliminating Syt1 from rods in Rho-iCre/Syt1^flfl^/Ai32 mice did not cause a statistically significant reduction in amplitude or charge transfer of rod-driven currents in the presence of strychnine (**Figures 7**, **8**). Time to peak latency was also not significantly altered (**Figure 8C**). OFF transient α cells receive stronger inputs from type 3 and 4 cone OFF bipolar cells that in turn receive direct rod input, but large optogenetic responses remained in OFF transient cells from mice lacking Syt1 in rods, averaging 1.0 ± 0.33 nA (n=3). This compared to optogenetic currents of OFF transient cells in control animals that averaged 1.6 ± 0.29 nA (n=6; p=0.79, unpaired t-test).

In addition to Syt1, rods can use Syt7 for a slow form of synaptic release and so we considered the possibility that this sensor might contribute to release in the absence of Syt1 (32). We bred floxed Syt7 mice (32) to generate Rho-iCre/Syt1^flfl^/Syt7^flfl^/Ai32 mice that lacked both Syt1 and Syt7 in rods. Simultaneous elimination of both Syt1 and Syt7 from rods did not reduce rod-driven optogenetic responses in Off RGCs, suggesting that Syt7 was not responsible for the responses to optogenetic stimulation of rods that remained in the absence of Syt1 in rods (**Figure 8**).

To test the requirement for synaptic transmission from cones when output from rods was blocked by elimination of Syt1, we examined responses of OFF ganglion cells in mice that had ChRh2 in rods and cones but lacked Syt1 in both. To do so, we crossed Rho-iCre/HRGP-Cre/Ai32 mice with floxed Syt1 mice (Syt1^fl/fl^) to generate conditional Syt1 knockouts that lacked Syt1 in both rods and cones. Eliminating Syt1 from both rods and cones completely abolished responses to optogenetic stimulation of photoreceptors (**Figures 7G**, **8**). This experiment shows that responses evoked by optogenetic stimulation of rods lacking Syt1 were not due to an unidentified Ca^2+^ sensor in rods and are consistent with a requirement for Syt1-mediated release from cones. While this does not eliminate the possibility of modest contributions from the tertiary pathway, these data show that large responses evoked by optogenetic stimulation of rods lacking Syt1 were entirely mediated by the secondary pathway.

To eliminate the secondary pathway and isolate direct transmission from rods to OFF bipolar cells, we tested Cx36 knockout mice that lack gap junctions between rods and cones (as well as lacking gap junctions between AII amacrine cells and ON-type cone bipolar cells) (33). Optogenetic stimulation of rods in Rho-iCre/Ai32 mice crossed with Cx36 knockout mice evoked modest responses (428 ± 414 pA, n=10 cells, 3 mice) with long latencies (40.7 ± 14.1 ms; **Figure 8**) in most OFF RGCs. Long latencies are more consistent with poly-synaptic inputs than direct inputs from cone OFF bipolar cells to RGCs. Two cells showed short latencies more consistent with direct inputs from OFF bipolar cells to OFF RGCs. One was an OFF sustained cell with a latency of 20 ms and peak amplitude of 580 pA. The other was an OFF transient cell with a latency of 15.5 ms and amplitude of 1.5 nA. The response of this latter cell is illustrated in **Figure 7F** (red trace). The black trace shows an example from a different OFF transient cell in the same retina that was more typical of the slow responses seen in other Cx36 KO RGCs. The finding that most OFF RGCs showed small and slow responses when primary and secondary pathways were eliminated suggests rod signals reached these cells through an indirect pathway, presumably involving amacrine cells. However, a subset of OFF cells show faster responses that are more likely due to direct OFF bipolar cell inputs into RGCs.

## Discussion

We combined pharmacology, knockouts, and optogenetics to analyze the pathways by which rods signals travel through the mouse retina. Blocking glycinergic synapses between AII amacrine and cone OFF bipolar cells with strychnine reduced optogenetic currents by ~1/3. This suggests that with optogenetic stimulation, ~1/3 of the rod input into the OFF pathway flows through inhibitory glycinergic synapses from AII amacrine cells to cone bipolar cells. This is a lower bound estimate since the activity of rod bipolar cells is nearly saturated by glutamate release in darkness (52) and so additional glutamate release evoked by optogenetic stimulation of rods can have only a limited impact on transmission to rod bipolar cells. Furthermore, picrotin, which is a component of picrotoxin, can inhibit glycine receptors so some inhibition of the primary pathway remained even in the absence of strychnine (53).

In the presence of strychnine to block the primary rod pathway, large, fast currents could be evoked by optogenetic stimulation of rods in both transient and sustained OFF α RGCs. This was true even after eliminating glutamate release from rods by genetic deletion of Syt1 and Syt7. Since eliminating glutamate release from rods should eliminate both primary and tertiary rod pathways, the large responses evoked by optogenetic stimulation of rods in the presence of strychnine must have arisen solely from the secondary pathway. Strong optogenetic stimuli may engage pathways that normally operate at higher light levels and so the secondary pathway may play a particularly prominent role in our experiments (4, 11).

With the primary pathway blocked by strychnine, the latency of optogenetic responses evoked by stimulation of rods was 4.5 ms longer in OFF RGCs than responses evoked by optogenetic stimulation of cones. Cones have an average membrane capacitance of 6.2 pF and input resistance of 0.53 GΩ (31) suggesting a membrane time constant of 3.3 ms. The added time needed for transmission of voltage changes through gap junctions to cones could potentially account for the longer latency of rod-driven responses traveling through the secondary pathway.

In the presence of strychnine to suppress the primary pathway, we inhibited the secondary pathway by inhibiting rod-cone gap junctions with MFA or by activating D2 receptors with quinpirole. We also tested Cx36 KO mice that lack gap junctions between rods and cones. Under these conditions, the tertiary rod pathway involving direct inputs from rods to cone OFF bipolar cells should be the major or sole remaining pathway. And under these conditions, optogenetic stimulation of rods generally evoked small, slow responses in OFF RGCs more consistent with poly-synaptic inputs than direct inputs from cone OFF bipolar cells. However, we saw fast responses in two RGCs from mice lacking Cx36 suggesting a subset receive direct contacts from OFF bipolar cells that in turn receive direct input from rods.

In mice lacking Cx36, the slow kinetics of responses evoked by optogenetic stimulation of rods suggested they arrive through a pathway that involves amacrine cells. One possible sign-conserving pathway from cone OFF bipolar cells would be from cone OFF bipolar cells →glutamatergic monopolar amacrine cells → OFF RGCs (54). Another possible way to achieve a sign-conserving pathway from cone OFF bipolar cells is serial inhibition (→ inhibitory amacrine cells → other inhibitory amacrine cells → OFF RGCs). Input into rod bipolar cells is intact in Cx36KO mice and so another possibility is a sign-inverting pathway from rod bipolar cells. In addition to contacting AII amacrine cells, rod bipolar cells also contact A17 and nNOS amacrine cells (55, 56). And while A17 cells make most of their synapses onto rod bipolar cells, they also make occasional connections with cone bipolar cells (57). Furthermore, synapses from A17 cells onto bipolar cells involve GABA_c_ receptors that may not have been fully blocked by picrotoxin used in most of our experiments, including those with Cx36 KO mice (58).

Anatomical evidence suggests that OFF bipolar cells provide greater direct input into transient OFF α cells than OFF sustained cells (27, 28). Consistent with this, we found one transient OFF RGC that exhibited particularly large fast currents when rods were stimulated optogenetically in retinas lacking Cx36. Rods make direct contact with types 3 and 4 cone OFF bipolar cells (8, 14–19) and types 3A and 4 cells provide most of the input into OFF transient α cells (27, 28, 59). The OFF transient cell that showed large fast responses consistent with strong direct input from cone OFF bipolar cells was similar in soma size and dendritic extent to other OFF transient cells, but subtypes of OFF ganglion cells can be difficult to distinguish without further careful study (e.g., OFF sustained and bursty-suppressed-by-contrast ganglion cells share a similar morphology) (60–63).

Recordings from individual rods showed that Syt7 contributes to synaptic release when stimulated with long depolarizing stimuli (32). Eliminating Syt7 from rods abolished this slow form of release but, surprisingly, had no effect on ERG b-waves and eliminating Syt1 alone from rods and cones was sufficient to abolish b-waves. Similarly, the present results showed that eliminating Syt1 from both rods and cones was sufficient to abolish responses evoked by optogenetic stimulation of rods, even with Syt7 intact. These data provide further support for the idea that Syt1 alone is responsible for mediating fast responses of rods. We hypothesize that Syt7 may play a modulatory role by slowly adjusting synaptic cleft levels of glutamate as rod membrane potential varies with light intensity.

How do our results on rod pathways compare to previously published studies? Using multielectrode arrays to study mouse retina, Seilheimer et al. (3) saw several responsive OFF cells under scotopic conditions in Cx36KO mice but no responsive OFF RGCs when they tested retinas lacking rod bipolar cells. This suggests that the primary pathway is essential for most scotopic OFF responses (3). Using whole cell recordings, Protti et al. (9) found that blocking rod bipolar cells with L-AP4 blocked scotopic responses in 17/18 OFF and ON/OFF ganglion cells in mouse retina, also suggesting an essential role for the primary rod pathway in mediating OFF responses (9). In both studies (3, 9), RGC responses were restored at higher intensities where it is thought that rod-cone gap junctions contribute more significantly (4). Contributions from the secondary rod pathway have been shown in humans by flicker ERG responses (12, 13) and there is evidence that the tertiary pathway provides minimal inputs to OFF parasol ganglion cells in primate retina (64). Jin et al. dissected the different rod pathways using a combination of knockout mice and pharmacology (11) and characterized the intensity ranges over which these different pathways operate. Their results showed that the primary pathway conveys low scotopic information, the secondary pathway operates at high scotopic levels, and the tertiary pathway contributes at mesopic levels. After blocking the primary pathway with L-AP4 and removing the secondary pathway by eliminating rod/cone gap junctions, the tertiary pathway remained capable of supporting robust responses in OFF cells. Jin et al. did not directly assess kinetics, but our results suggest that much of this tertiary pathway involves slow kinetics and passage through intermediary amacrine cells with only a subset of RGCs receiving fast direct inputs from cone OFF bipolar cells.

Pasquale et al. (65) found that GNAT2 KO mice lacking cone light responses crossed with Cx36 KO mice lacking functional gap junctions retained a surprising degree of contrast sensitivity at high temporal frequencies. Cx36-independent, rod-driven responses must arise from either primary or tertiary pathways. Pasquale et al. argued that the intensity range was too high to be mediated by the primary rod pathway, but there is evidence that the primary rod pathway can contribute over intensities extending into the mesopic range (10). Contacts between rods and cone ON bipolar cells might also contribute (22). The slow responses that we saw in most OFF cells after blocking gap junctions would not have the capability to transmit high temporal frequency information *via* the tertiary pathway, but a subset of OFF transient cells may be specialized to carry this sort of information.

In summary, our data suggest that in addition to significant contributions from the primary rod pathway, much of the remaining input into OFF α ganglion cells of the mouse retina involves transmission through gap junctions to cones. With optogenetic stimuli, the tertiary OFF pathway provides slow indirect input to most OFF RGCs but a subset of RGCs showed fast responses consistent with direct input from cone OFF bipolar cells. These fast direct connections may be particularly important for informing the brain about fast rod-mediated responses under mesopic conditions (65).

## Data availability statement

The raw data supporting the conclusions of this article will be made available by the authors, without undue reservation.

## Ethics statement

The animal study was reviewed and approved by Institutional Animal Care and Use Committee of the University of Nebraska Medical Center.

## Author contributions

WT designed the project. AS and WT carried out experiments. AS and WT analyzed the data. WT produced figures and wrote the manuscript. All authors contributed to the article and approved the submitted version.
